# The anti-fibrotic effect of human fetal skin-derived stem cell secretome on the liver fibrosis

**DOI:** 10.1186/s13287-020-01891-5

**Published:** 2020-09-03

**Authors:** Xia Yao, Jing Wang, Jiajing Zhu, Xiaoli Rong

**Affiliations:** 1 Department of Anesthesiology, The Affiliated Hospital of Changchun University of Chinese Medicine, 1478 Gongnong Road, Changchun, 130021 Jilin China; 2 Department of Gynecology, The Affiliated Hospital of Changchun University of Chinese Medicine, 1478 Gongnong Road, Changchun, 130021 Jilin China; 3 Department of Radiology, The Third Hospital of Jilin University, 126 Xiantai St., Changchun, 130033 Jilin China; 4 Department of Clinical Laboratory, The Affiliated Hospital of Changchun University of Chinese Medicine, 1478 Gongnong Road, Changchun, 130021 Jilin China; 5 Neuroregeneration and Stem Cell Programs, Institute for Cell Engineering, The Johns Hopkins University School of Medicine, Baltimore, MD 21205 USA

**Keywords:** hFSSC, Secretome, Liver fibrosis, TGF-β/Smad

## Abstract

**Background:**

Liver fibrosis resulting from chronic liver injury is one of the major causes of mortality worldwide. Stem cell-secreted secretome has been evaluated for overcoming the limitations of cell-based therapy in hepatic disease, while maintaining its advantages.

**Methods:**

In this study, we investigated the effect of human fetal skin-derived stem cell (hFSSC) secretome in the treatment of liver fibrosis. To determine the therapeutic potential of the hFSSC secretome in liver fibrosis, we established the CCl_4_-induced rat liver fibrosis model and administered hFSSC secretome in vivo. Moreover, we investigated the anti-fibrotic mechanism of hFSSC secretome in hepatic stellate cells (HSCs).

**Results:**

Our results showed that hFSSC secretome effectively reduced collagen content in liver, improved the liver function and promoted liver regeneration. Interestingly, we also found that hFSSC secretome reduced liver fibrosis through suppressing the epithelial-mesenchymal transition (EMT) process. In addition, we found that hFSSC secretome inhibited the TGF-β1, Smad2, Smad3, and Collagen I expression, however, increased the Smad7 expression.

**Conclusions:**

In conclusions, our results suggest that hFSSC secretome treatment could reduce CCl_4_-induced liver fibrosis via regulating the TGF-β/Smad signal pathway.

## Introduction

Liver fibrosis is a wound healing response generated against chronic or iterative liver injury [1]. Recent evidence suggests that stem cell-based liver fibrosis treatment can be mediated through paracrine effects [2, 3]. The exclusive use of stem cell-secreted secretome has been evaluated for overcoming the limitations of cell-based therapy, while maintaining its advantages to their parent cells [4]. It included extracellular vesicles and other soluble proteins or biologically active molecules. In addition, previous studies have indicated that human bone marrow mesenchymal stem cell-derived exosomes and other stem cell-derived secretome can reduce liver fibrosis [5, 6].

Previous studies have indicated that the features of fetal tissue cells facilitate engraftment in vivo and may provide preferred effects against diseases difficult to treat [7]. Since 1928, hundreds of clinical trials using various types of fetal transplants have been performed worldwide [8–10]. Moreover, recent studies have demonstrated that human fetal stem cells (FSCs) have a great growth promoting potential, which benefited to the tissue regeneration and cell therapy [11, 12]. In comparison to other mesenchymal stem cell (MSCs), FSCs are easier to culture and more readily proliferate and less likely to be rejected by transplant recipients, as these cells are less antigenic [8]. Although there remain still ethical and social issues with respect to the clinical use of fetal tissue, fetal stem cell secretome transplantation may overcome these problems and have more perspectives on hepatic disease treatment.

In our previous study, we have successfully isolated and identified hFSSCs [13]. Interestingly, we found hFSSC secretome has great ability to control and balance the collagen formation in skin tissue [13]. Therefore, we hypothesized that hFSSCs have a potential to reduce the collagen formation in liver fibrosis, whereas liver fibrosis is caused by over-abundance of collagen. We further analyzed the effects of hFSSC secretome on live fibrosis in vitro and investigated the mechanism of hFSSC secretome and TGF-β/Smad pathway involvement on anti-fibrosis. Our preliminary results provide the first evidence that hFSSC secretome effectively reduce liver fibrosis through the TGF-β/Smad pathway. We believe that the hFSSC secretome as an acellular regenerative therapy and approaches can provide great potential for the treatment of liver fibrosis.

## Materials and methods

### Cell culture

hFSSCs and human umbilical cord mesenchymal stem cell (hUCMSCs) were provided and extracted by our previous study [13]. HSCs were purchased from the Chinese Academy of Medical Sciences, China. In brief, hFSSCs, hUCMSCs, and HSCs were cultured in high glucose DMEM (Gibco, Grand island, USA) supplemented with 500 U/ml penicillin and 500 μg/ml streptomycin (Invitrogen, Shanghai, China), and 10% FBS (Gibco, Grand island, USA) at 37 °C, with saturated humidity and 5% CO_2_. hFSSCs and hUCMSCs at the P5 were used for this study. hFSSC and hUCMSC secretome was collected as reported in our previous study [13]. Briefly, cells were cultured and reached 70~80% confluence, then placed in serum-free medium (SFM; Invitrogen, Shanghai, China), added with 5 ml of 200 mM l-glutamine solution (Invitrogen, Shanghai, China) in 500 ml SFM prior to use, and incubated in 5% CO_2_ in a humidified condition. After being cultured 24 h, the conditioned medium (CM) was collected and centrifuged to purify for 10 min at 4 °C, 4000*g*. Next, 10-ml conditioned medium was re-centrifuged with Amicon Ultra Centrifugal Filters (Millipore Corp, Billerica, MA, USA) at 4 °C, 4000 g, 2 h. At last, 300~500 μl supernatant solution was collected as cell-free secretome each time. The protein content was measured using the BCSA kit (Thermo Scientific, Rockford, IL, USA) according to the manufacturer’s instruction.

### CCl_4_-induced liver fibrosis in rats

Liver fibrosis was induced in Sprague Dawley (SD) rats (8-week-old, female, 200 g). All protocols and procedures were approved by the Animal Experiment Ethic Committee of Changchun University of Traditional Chinese Medicine (Approval NO. XW201903167). All experimental procedures were in accordance with the Chinese legislation regarding experimental animals. Detailed procedures for CCl_4_-induced have been described in our published studies [6]. Briefly, rats were administered with an intraperitoneal injection of 30% CCl_4_, 3 ml/kg body weight twice weekly in olive oil. After 8 weeks, CCl_4_-treated rats were randomly assigned into three groups (*n* = 10 rats, tail vein injection/weekly): PBS group (1 ml), hUCMSC secretome group (250 μg, 1 ml), and hFSSC secretome group (250 μg, 1 ml). After 4 weeks, liver tissue and serum were collected. Livers were divided into two parts of preservation in 10% formalin and freezing at − 80 °C.

### Histopathological analysis

Liver tissues were processed for paraffin embedding by slicing into 4-μm sections. Liver sections were stained with hematoxylin and eosin (H&E), Masson, and Sirius red according to standard protocols. We selected the liver section fields randomly to analyze the liver fibrosis. The percentage of collagen-stained area was calculated via Image-Pro Plus. Immunohistochemistry (IHC) was measured with the Kit (Maixin KIT-9710, Fuzhou, China) in accordance with the manufacturer’s instructions. In brief, the liver sections were deparaffinized, rehydrated, and incubated in a 99 °C water bath for 15 min. Then, the sections were incubated with 3% H_2_O_2_ for 15 min and blocked with 10% normal goat serum (Sigma, USA) for 1 h at 37 °C, following with the incubation of primary antibody against PCNA (ab15497, 1:500 dilution, Abcam, Cambridge, UK), α-SMA (ab5694, 1:500 dilution, Abcam, Cambridge, UK), and HNF-4α (ab219610, 1:500 dilution, Abcam, Cambridge, UK) overnight at 4 °C. Next, sections were incubated with biotinylated goat-anti-rabbit IgG antibody, added with diaminobenzidine solution for 15 min at 37 °C, then incubated with avidin peroxidase reagent, and hematoxylin for counterstaining. Lastly, slides were photographed using an optical microscope (Olympus, Tokyo Metropolitan, Japan). We used 10 random fields per section and 10 sections in total (*n* = 10 rats) for quantification of IHC results. The IHC results were calculated via Image-Pro Plus.

### Biochemical analysis

The serum levels of alanine aminotransferase (ALT), aspartate aminotransferase (AST), total protein (TP), total bilirubin (TBIL), alkaline phosphatase (ALP), and gamma glutamyl transpeptidase (γ-GT) were assessed using the Automated Biochemical Analyzer (AU-680, Beckman, California, USA) according to the procedure. Liver homogenate (10%, w/v) was prepared by homogenizing the right lobe of liver on ice in 150 mM Tris-HCl buffered saline (pH 7.2) using a polytron homogenizer (PT3100D; Kinematical, Lucerne, Switzerland). The levels of malondialdehyde (MDA) and hydroxyproline (Hyp) in liver tissue were measured using kits (NanJing JianCheng Bio., Nanjing, China) according to the manufacturer’s instructions.

### Quantitative real-time PCR (RT-qPCR)

HSCs were co-cultured with either PBS, hUCMSC secretome, or hFSSC secretome (5 ng/ml) for 48 h before samples were collected for mRNA extraction. Total RNA was isolated from HSCs using Trizol reagent (Invitrogen, Shanghai, China) according to the manufacturer’s protocol. Then, 1 μg total RNA was reverse-transcribed to give cDNA, which was used as the template, and combined with standard SYBR premix Ex Taq (Invitrogen, Shanghai, China) on the qPCR Detection System (Roche, Basel, Switzerland), and experiments were conducted in triplicate. The levels of EMT-related genes (E-cadherin, Snail1, Vimentin, FSP1, and α-SMA), TGF-β/Smad signaling pathway-related genes (TGF-β1, Smad2, Smad3, Smad7 and Collagen I), and the internal standard GAPDH mRNA were measured by RT-qPCR. The primers are listed in Table S1, and GAPDH served as the internal control. All reactions were performed in triplicate, and the data were analyzed using the 2^−ΔΔCt^ method.

### Immunofluorescence (IF) staining

When HSCs reached 60~70% confluence on 24-well plates, they were cultured with either PBS, hUCMSC secretome, or hFSSC secretome (5 ng/ml) for 48 h. Next, HSCs were incubated with 4% paraformaldehyde at room temperature for 10 min and then incubated with 1% bovine serum albumin (BSA, Biosharp, Wuhan, China) for 30 min. Cells were incubated with a primary antibody against α-SMA (ab5694, 1:100 dilution, Abcam, Cambridge, UK) for 1 h, followed by incubation with a secondary antibody (goat anti-rabbit IgG, ab15007, 1:500 dilution, Abcam, Cambridge, UK) for 30 min at room temperature. Rhodamine phalloidin (Thermal Scientific, Waltham, USA) was stained for cytoskeleton. The nuclei were labeled with DAPI (Thermal Scientific, Waltham, USA). Fluorescent images were captured using an EVOS Cell Imaging System (Thermo Scientific, Waltham, USA).

### Western blotting

HSCs were co-cultured with either PBS, hUCMSC secretome, or hFSSC secretome (5 ng/ml) for 48 h before samples were collected for protein extraction. Protein samples were mixed with SDS sample buffer and heated to 95 °C for 10 min, followed by separation on SDS-polyacrylamide gels. Resolved proteins were electro-blotted onto nitrocellulose membrane and probed with antibodies against TGF-β1 (ab92486), Smad2 (ab40855), Smad3 (ab40854), Smad7 (ab216428), Collagen I (ab90395), and β-actin (ab5694), (1:1000 dilution, Abcam, Cambridge, UK) overnight at 4 °C (1:1000 dilution, Abcam, Cambridge, UK). Nitrocellulose membranes were then incubated with a secondary antibody, HRP-conjugated goat anti-rabbit IgG (ab15007), at room temperature for 2 h, and visualized by chemiluminescent detection according to the manufacturer’s instructions (Immobilon western chemiluminescent HRP substrate, Millipore, Massachusetts, USA).

### Statistical analysis

Statistical analysis was performed using GraphPad Prism Version 6. One-way ANOVA with Dunnett’s multiple comparisons test was used to test for statistically significant differences. All quantitative data are expressed as mean ± SD for at least three independent experiments, and *p* < 0.05 was considered to be statistically significant.

## Results

### hFSSC secretome reduced CCl_4_-induced liver fibrosis in rats

**Fig. 1:**
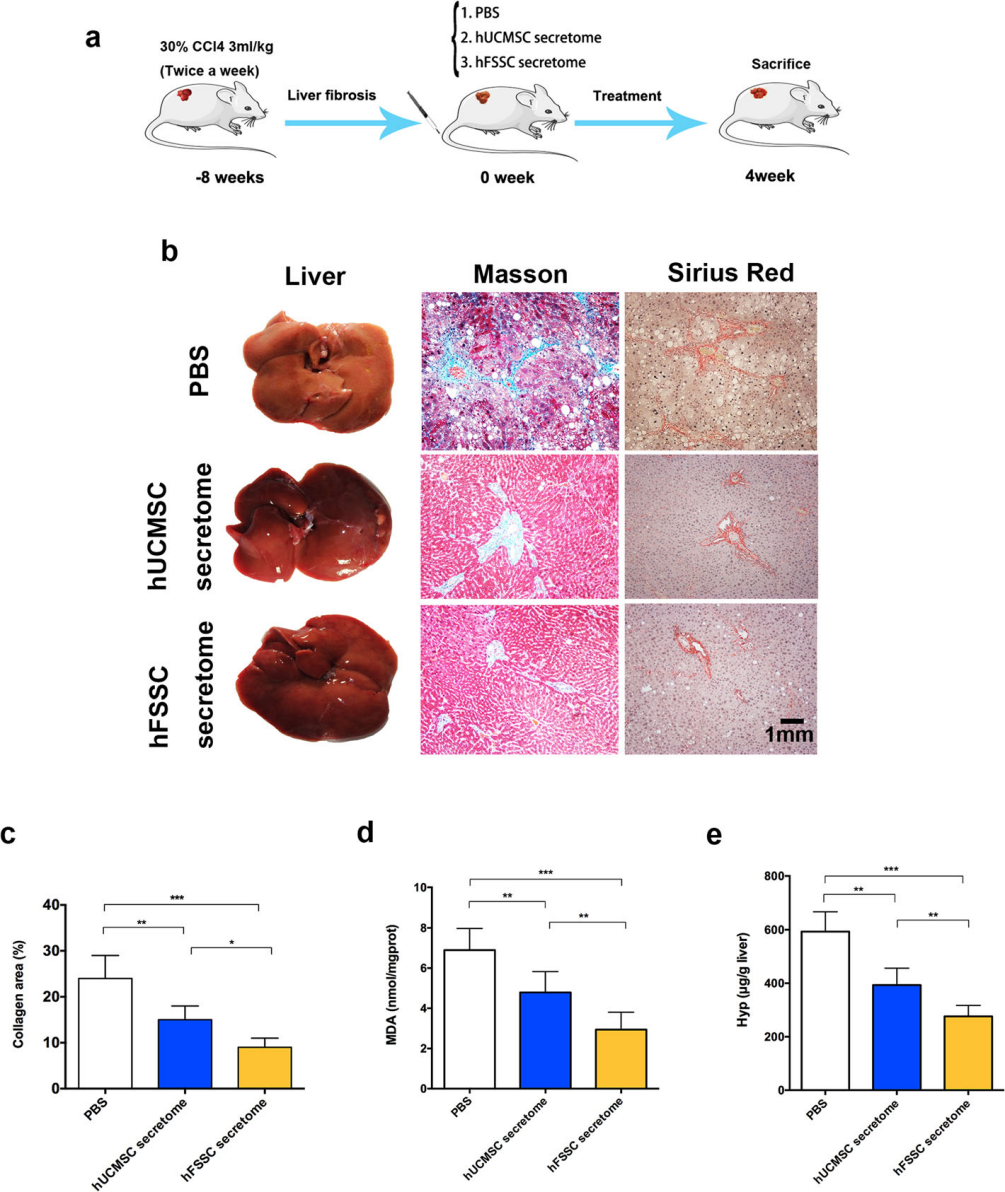
hFSSC secretome reduced liver fibrosis in rats. **a** Experimental design. **b** The representative images of gross morphology, Masson, and Sirius red staining analysis of liver. Bar = 1 mm, *n* = 10 rats. **c** Quantitative analysis of the collagen area percentage at 4 weeks. **d** Quantitative analysis of hepatic MDA and Hyp content. *p < 0.05, **p < 0.01, ***p < 0.001. *n* = 10; mean ± SD

To explore the effect of hFSSC secretome on liver fibrosis, we used CCl_4_-induced liver fibrosis model in rats (Fig. 1a). Compared to the PBS group, gross morphology changes obviously in the hFSSC secretome group, including less fibrous nodular and more ruddy on the surface, more uniform surface, and soft texture (Fig. 1b). After 4 weeks’ treatment, histopathological analysis using Masson and Sirius red staining indicated that the collagen area percentage in the hFSSC group (9.2%) was significantly reduced, compared to the other two control groups (24.3% in the PBS group and 14.9% in the hUCMSC secretome group, Fig. 1b and c, *p* < 0.05). Furthermore, we detected the MDA (a marker for oxidative stress and liver cell injury) and Hyp (a main component in collagen tissue) content in the liver tissue. We found that the level of MDA and Hyp in the hFSSC secretome group was significantly lower than the other two control groups (Fig. 1d and e *p* < 0.01). These findings suggest that hFSSC secretome effectively reduced CCl_4_-induced liver fibrosis in rats.

### hFSSC secretome reduced liver fibrosis through suppressing the EMT

**Fig. 2:**
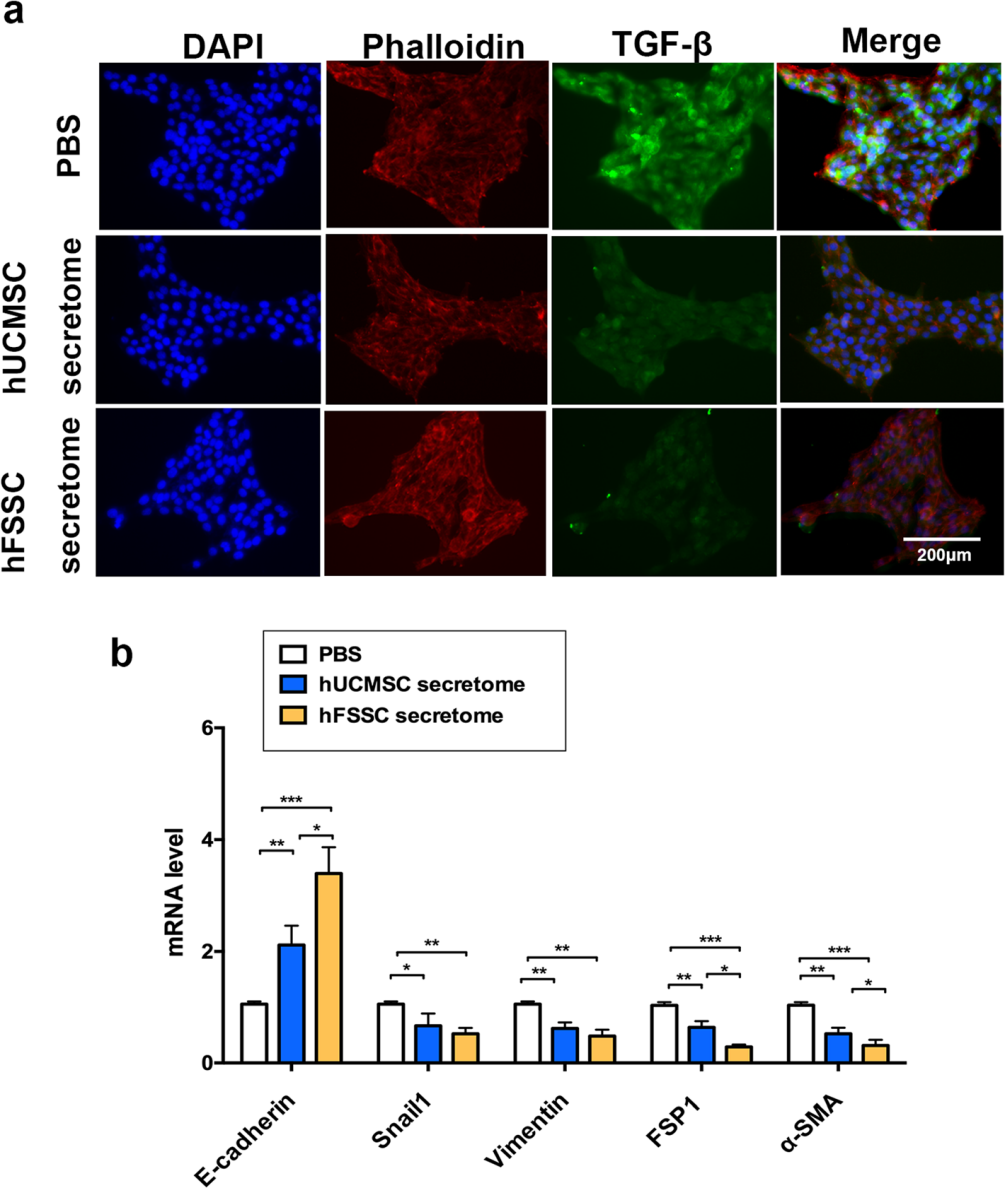
hFSSC secretome reduced liver fibrosis through suppressing the EMT process. **a** Representative images of immunofluorescence staining performed for TGF-β in HSCs. Bar = 200 μm. **b** Relative mRNA expression levels of EMT-related gene, included of E-cadherin, Snail1, Vimentin, FSP1, and α-SMA in HSCs. EMT, epithelial–mesenchymal transition; FSP1, fibroblast-specific protein 1; α-SMA, alpha smooth muscle actin. *p < 0.05, **p < 0.001, ***p < 0.0001. *n* = 10, mean ± SD

To further verify the roles of hFSSC secretome in the pathogenesis of liver fibrosis, we performed immunofluorescence staining of TGF-β in HSCs, as TGF-β1 is considered as a crucial mediator in tissue fibrosis and HSCs are one of the major effector cells in liver fibrosis. We found that the hFSSC secretome group reduced fluorescence intensity observably, compared to the other two control groups (Fig. 2a). In further study, we explore the effect of hFSSC secretome on EMT, and RT-qPCR analysis was used to examine the expression of EMT-related indicators (E-cadherin, Snail1, Vimentin, FSP1, and α-SMA in HSCs). Interestingly, our results found that hFSSC secretome treatment increased the epithelial marker of E-cadherin expression, while decreasing the transcription factors of Snail and mesenchymal marker (Vimentin, FSP1 and α-SMA) expression, compared to the PBS group (Fig. 2b, *p* < 0.05). Meanwhile, we also examined hFSSC secretome increased E-cadherin and decreased FSP1 and α-SMA expression compared to the hUCMSC secretome group (Fig. 2b, *p* < 0.05). These results suggest that hFSSC secretome reduced liver fibrosis through suppressing the EMT.

### hFSSC secretome improved liver functionality and promoted liver regeneration

**Fig. 3:**
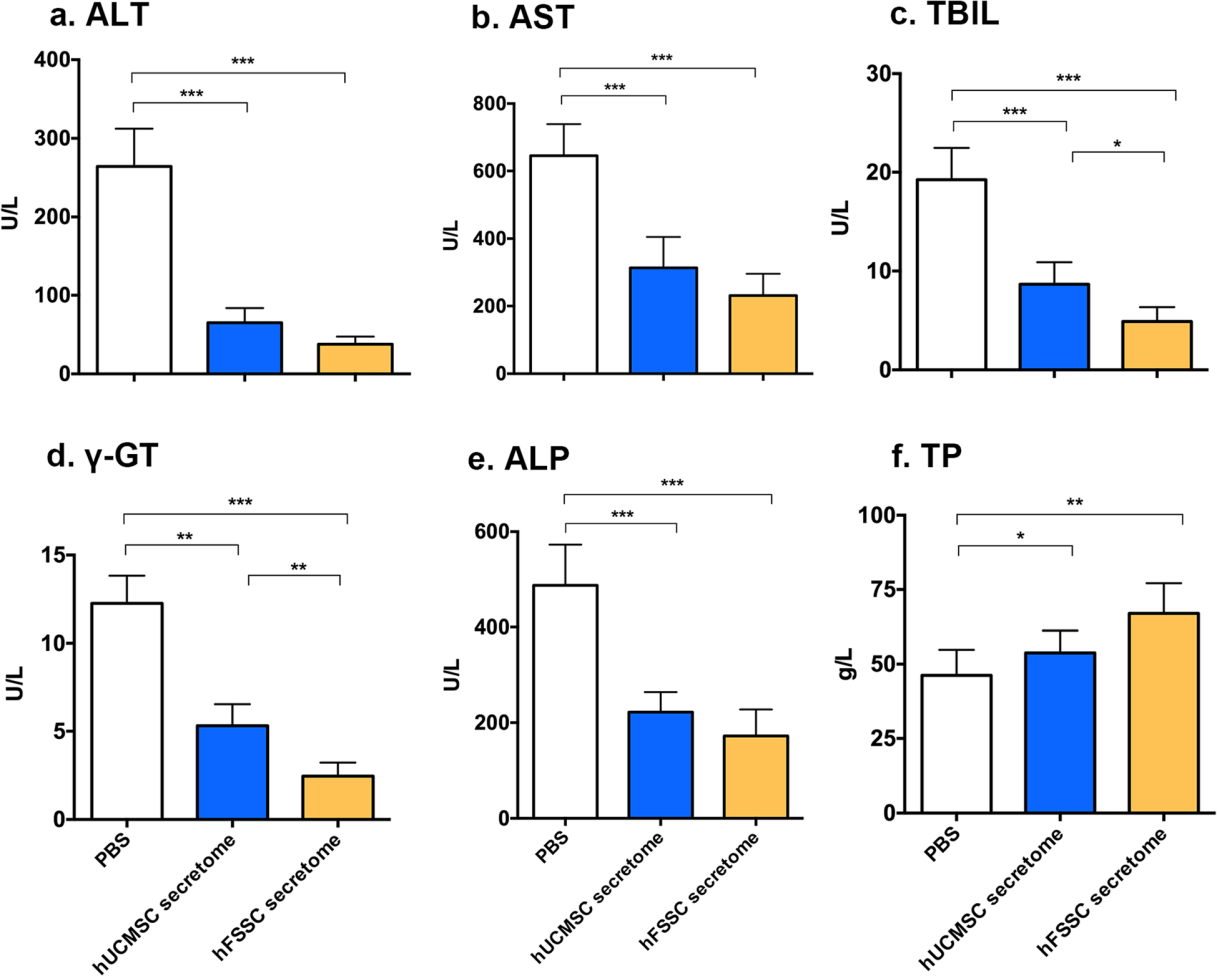
hFSSC secretome improves liver function in serum biochemical parameters. **a** ALT, alanine aminotransferase. **b** AST, aspartate aminotransferase. **c** TBIL, total bilirubin. **d.** γ-GT, gamma glutamyl transpeptidase. **e** ALP, alkaline phosphatase. **f** TP, total protein. *p < 0.05, **p < 0.01, ***p < 0.001. *n* = 10, mean ± SD

To explore the effect of hFSSC secretome on liver functionality, we performed the biochemical analyses. In comparison to the PBS group, hFSSC secretome group significantly reduced serum levels of ALT, AST, TBIL, γ-GT, and ALP (Fig. 3a–e, *p* < 0.05). However, the serum level of TP in hFSSC secretome group was higher than that in PBS group (Fig. 3f, *p* < 0.05). In addition, the hFSSC secretome group significantly reduced the serum levels of TBIL and γ-GT compared to the hUCMSC secretome group (Fig. 3c and d, *p* < 0.05). These results suggest that hFSSC secretome effectively improved liver functionality.

**Fig. 4:**
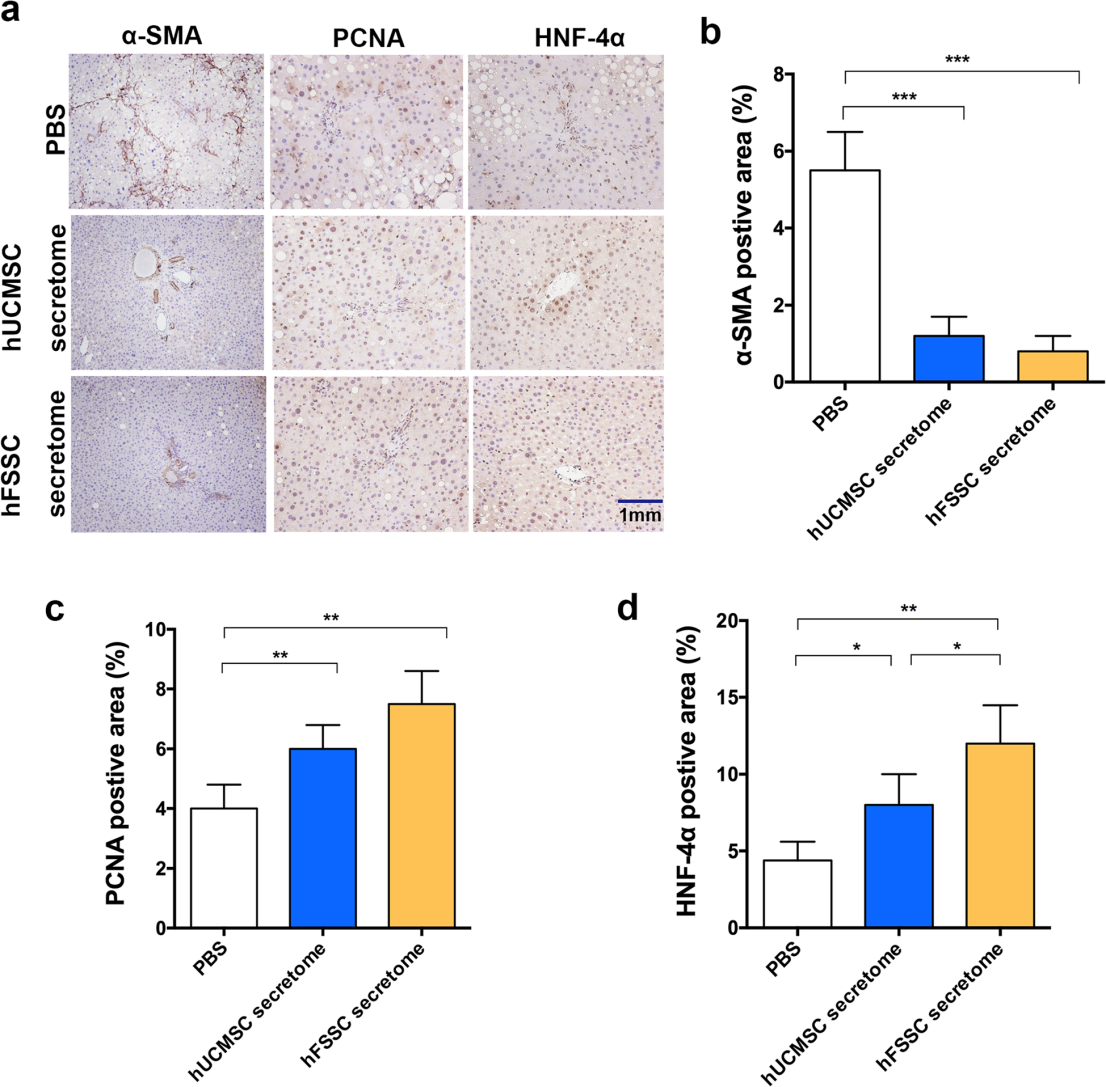
Histological analysis of hFSSC secretome reduced liver fibrosis and promoted liver regeneration. **a** Photomicrographs of liver tissue sections showing IHC staining for α-SMA, PCNA, and HNF-4α. Brown cells represent the positive expression, bar = 1 mm. **b**–**d** The quantification of α-SMA, PCNA, and HNF-4αpositive cells area. *p < 0.05, **p < 0.01, ***p < 0.001. *n* = 10, mean ± SD

Next, we performed IHC to assess the effects of the hFSSC secretome on the liver. α-SMA is an important indicator of the occurrence and development of hepatic fibrosis. IHC results showed that the percentage of α-SMA-positive area in hFSSC secretome group (0.82%) was significantly decreased compared to the PBS group (5.51%, Fig. 4a and b, *p* < 0.001). PCNA is a crucial indicator of cell proliferation. IHC results showed that the percentage of PCNA positive area in hFSSC secretome group (4.13%) was significantly increased compared to the PBS group (7.48%, Fig. 4a and c, *p* < 0.01). Consist with the above results, the percentage of HNF-4α-positive area was significantly increased in the hFSSC secretome group (12.1%), compared to the PBS group (4.5%, *p* < 0.01) as well as the hUC-MSCs group (8.2%, Fig. 4a and d, *p* < 0.05). The histological results indicated that hFSSC secretome effectively delayed the progression of liver fibrosis and promoted the liver regeneration.

### hFSSC secretome regulate the TGF-β/Smad signal pathway

**Fig. 5:**
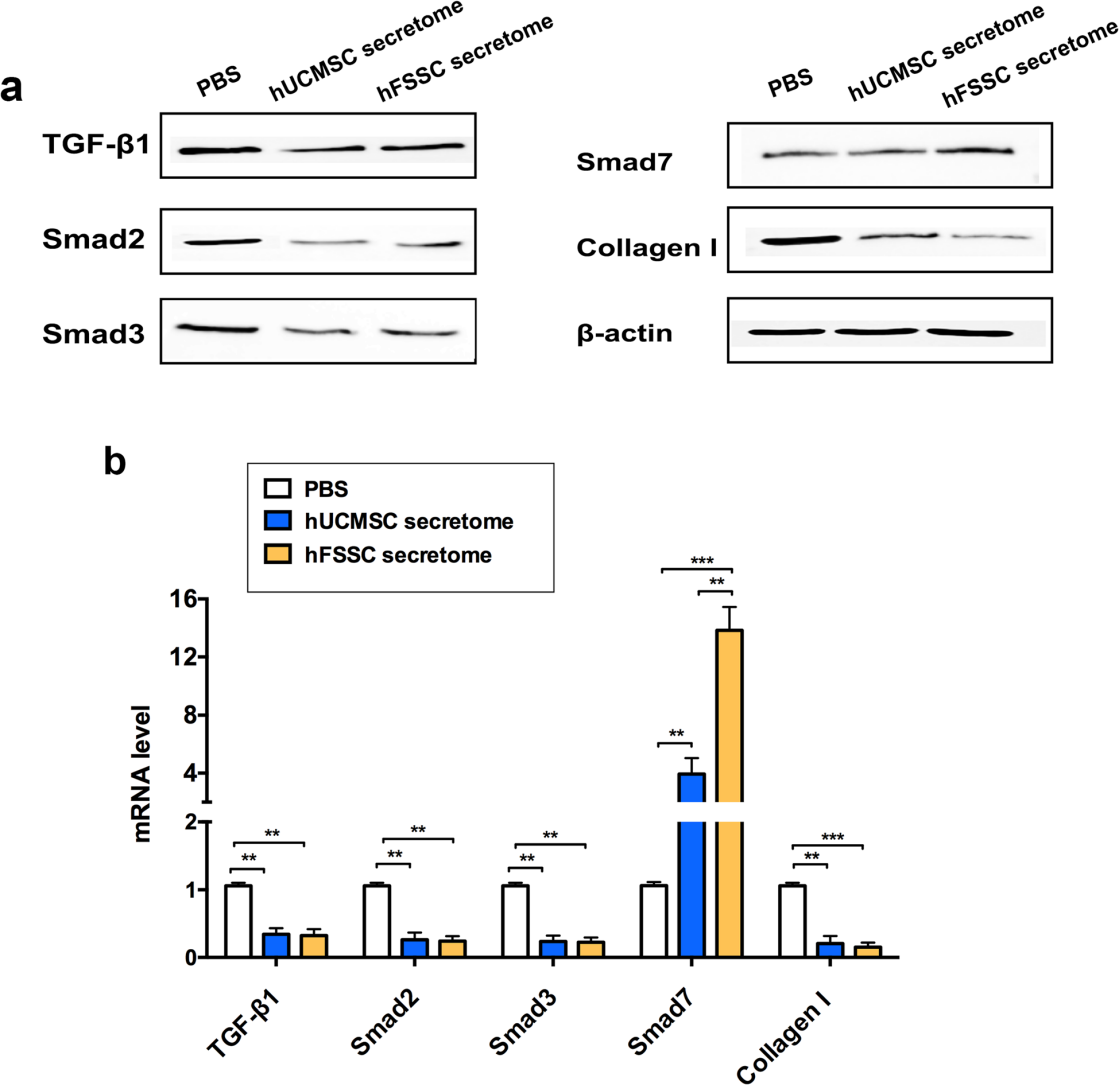
hFSSC secretome inhibited the TGF-β/Smad signaling pathway in HSCs. **a** Representative western blotting analysis the expression of TGF-β1, Smad2, Smad3, Smad7, and Collagen I in HSCs. **b** Relative mRNA expression levels of TGF-β1, Smad2, Smad3, Smad7, and Collagen I in HSCs. **p < 0.001, **p < 0.001. *n* = 3, mean ± SD

**Fig. 6:**
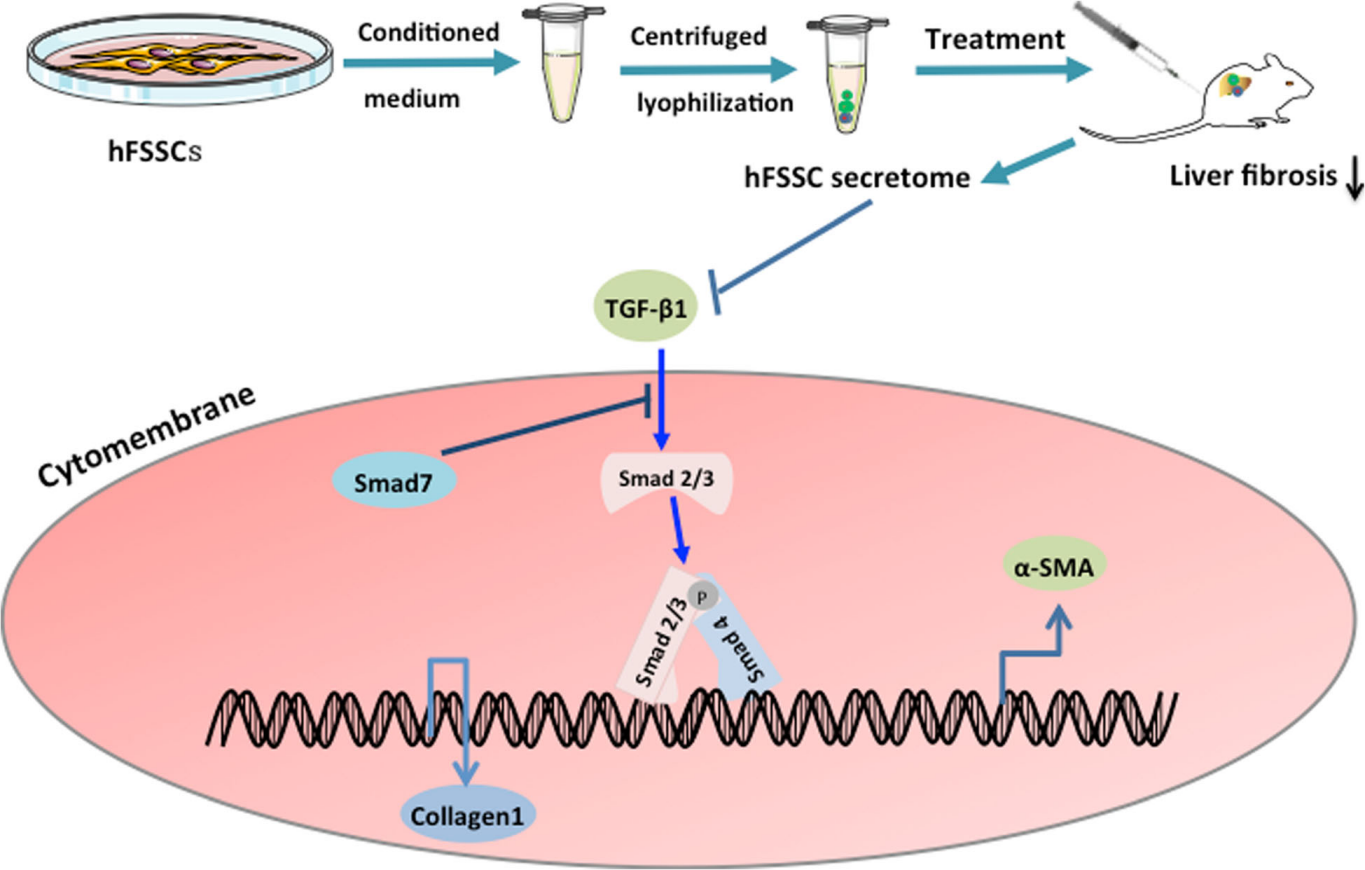
hFSSC secretome reduced liver fibrosis by regulating the TGF-β/Smad signaling pathway. hFSSC secretome inhibited TGF-β1expression and activated Smad7 expression which are activated by the binding of the TGF-β super family to the cell surface receptors. TGF-β1-mediated tissue fibrosis via Smad2/Smad3 which the two major downstream regulator, while Smad7 serves as a negative feedback regulator of TGF-β1/Smad pathway, thereby protecting against TGF-β1-mediated fibrosis. Furthermore, hFSSC secretome decreased the collagen I expression, thereby reducing liver fibrosis

To investigate the underlying mechanism of the effect of hFSSC secretome on liver fibrosis, we performed the Western blot and RT-qPCR to analyze the expression of TGF-β1, Smad2, Smad3, Smad7, and Collagen I in HSCs, as it is one of the major effector cells in liver fibrosis. We found that TGF-β1, Smad2, Smad3, and Collagen I expression was significantly decreased in hFSSC secretome group, compared to that of the PBS group (Fig. 5a and b, *p* < 0.001). However, we detected the Smad7 was significantly increased in the hFSSC secretome group, compared to that of the other two control groups (Fig. 5a and b, *p* < 0.01). Smad7 serves as a negative feedback regulator of TGF-β1/Smad pathway, thereby protecting against TGF-β1-mediated fibrosis (Fig. 6). These results suggest that hFSSC secretome effectively reduced liver fibrosis via regulating the TGF-β/Smad signal pathway (Fig. 5).

## Discussion

In this study, our results demonstrated that hFSSC secretome reduce liver fibrosis in rats. Moreover, our research illustrated that hFSSC secretome reduce liver fibrosis through suppressing the EMT and regulating the TGF-β/Smad signal pathway in HSCs.

Recent evidence indicates that the mesenchymal stem cell secretome as an acellular regenerative therapy for liver disease [5, 14]. The use of MSC secretome has been shown to have anti-fibrotic effects [14]. Moreover, IV injection of hucMSC-EVs decreased liver fibrosis, reduced apoptosis, and mitigated liver damage induced by CCl_4_ in mice [15, 16]. In our study, we found that hFSSC secretome effectively reduced CCl_4_-induced liver fibrosis and improved liver functionality in rats (Fig. 1). Moreover, hFSSC secretome treatment is more effective than that of hUCMSC secretome in resolving fibrosis, such as some indicators including collagen area, MDA, Hyp, TBIL, and γ-GT. The reasons that differences were found between hUCMSCs and hFSSC secretome may be reported that hFSSC secretome contains more biological activity factors than hUCMSC secretome [13]. Our studies indicated that hFSSC secretom is an attractive emerging option for therapeutic applications as a therapeutic strategy for liver fibrosis.

Previous study have showed that mesenchymal stem cell secretome inhibit HSC activation and promoted liver regeneration [17–19]. HSCs stimulate the production of a large amount of collagen fibers to form liver fibrosis. Moreover, positive expression of α-SMA can serve as a marker for HSC activation [20]. Our results demonstrate that hFSSC secretome decreased α-SMA expression. PCNA is a crucial indicator of cell proliferation, and HNF-4α is the master regulator of hepatic cell differentiation [15]. Our study demonstrates that the percentage of PCNA and HNF-4α-positive area was significantly increased after hFSSC secretome treatment. These results confirmed that hFSSC secretome promotes liver regeneration while reducing fibrosis. Despite our results confirming that hFSSC secretome had significantly reduced liver fibrosis and caused no detectable immunological responses, their exact mechanisms of action need further exploration. Moreover, study has reported that type 2 EMT is associated with tissue regeneration and organ fibrosis. After CCl_4_ injury in liver, inflammatory cytokines or chemical substances stimulate EMT to produce new collagen fibroblast pools to repair injury [5]. In present study, we demonstrated that hUCMSC secretome reduced liver fibrosis through suppressing the EMT process.

The MSC secretome secreted some growth factors and cytokines, such as hepatocyte growth factor (HGF), transforming growth factor beta isoform 3 (TGF-β3), and tumor necrosis factor-alpha (TNF-α), and IL-10 can modulate cell signaling and processes involved in fibrogenesis and can attenuate liver fibrosis [5, 21]. Recently, fetal mesenchymal stem cell functional secretome analysis illustrates that 737 ± 80 protein identifications was obtained from the amniotic fluid-mesenchymal stem cell (AF-MSC) secretome; interestingly, it reveals that Annexin-A1 is important paracrine factor in hepatic regeneration [22]. However, previous study demonstrated that hFSSC presented with the characteristics of both MSC and embryonic stem cell (ESC) [13]. These may be the partial reason that hFSSC secretome can reduce liver fibrosis and has better effects than hUCMSC secretome in some indictors.

TGF-β1/Smad pathway is an important pathogenic mechanism in tissue fibrosis [23, 24]. Studies have shown that TGF-β1 is considered as a crucial mediator in tissue fibrosis and causes tissue scarring largely by activating its downstream small mother against decapentaplegic (Smad) signaling [25]. However, different TGF-β signalings play different roles in fibrogenesis [24]. TGF-β1 directly activates Smad signaling which triggers pro-fibrotic gene overexpression [26]. Increasing studies have demonstrated that dysregulation of TGF-β1/Smad pathway was an important pathogenic mechanism in tissue fibrosis [27]. Smad2 and Smad3 are the two major downstream regulator that promote TGF-β1-mediated tissue fibrosis, while Smad7 serves as a negative feedback regulator of TGF-β1/Smad pathway, thereby protecting against TGF-β1-mediated fibrosis [24]. Our findings proved that hFSSC secretome effectively reduced TGF-β1, Smad2, Smad3, and Collagen I expression, moreover increasing Smad7 expression. It indicated that hFSSC secretome effectively reduced liver fibrosis via regulating the TGF-β/Smad signal pathway (Fig. 6).

In conclusion, we successfully investigated the role of hFSSC secretome on cutaneous liver fibrosis. Our results demonstrated that hFSSC secretome can exert promoting effect of liver fibrosis via regulating the TGF-β/Smad signal pathway.

## Supplementary information

**Additional file 1.** Table S1. Primers used for qRT-PCR

## Data Availability

The datasets used and/or analyzed during the present study are available from the corresponding author on reasonable request.

## Abbreviations

hFSSC: Human fetal skin-derived stem cell
MSCs: Mesenchymal stem cells
hUCMSCs: Human umbilical cord mesenchymal stem cell
HSCs: Hepatic stellate cells
ALT: Alanine aminotransferase
AST: Aspartate aminotransferase
TBIL: Total bilirubin
γ-GT: Gamma glutamyl transpeptidase
ALP: Alkaline phosphatase
TP: Total protein
Hyp: Hydroxyproline
MDA: Malondialdehyde
RT-qPCR: Quantitative real-time PCR
α-SMA: Alpha-smooth muscle actin
HGF: Hepatocyte growth factor
EMT: Epithelial to mesenchymal transition
TNF-α: Tumor necrosis factor-alpha
ESC: Embryonic stem cell
FSP1: Fibroblast-specific protein 1
