# Supplementary material for: The anti-fibrotic effect of human fetal skin-derived stem cell secretome on the liver fibrosis
Source: Stem Cell Res Ther. 2020 Sep 3;11:379. doi: 10.1186/s13287-020-01891-5 (PMC7650526; doi:10.1186/s13287-020-01891-5)
Supplement: Supplementary file 1 — Additional file 1. Table S1. Primers used for qRT-PCR [file 13287_2020_1891_MOESM1_ESM.docx]

**Table S1. Primers used for** **qRT-PCR**

| Gene | Primers | | Sequences | Product size (bp) |
| --- | --- | --- | --- | --- |
| GAPDH | Forward | | CCGAGACATCAAGGAGAAG | 151 |
|  | Reverse | | GTAGTTTCGTGAATGCCGCA |  |
| TGFβ1 | Forward | | GGACGCCGTAAGTGCTTTGA | 143 |
|  | Reverse | | GGACGAACGACTAGGTGTAG |  |
| Smad2 | Forward | | CCCTCCCAGAAGACCTACC | 138 |
|  | Reverse | | GGCACAAACACGCACCTCA |  |
| Smad3 | Forward | | GCTAACCCTTCGCTCCGT | 125 |
|  | Reverse | | GCTTCCTCCTCTTTATCAG |  |
| Smad7 | Forward | | CTCAGAACCTGCTTATCAA | 132 |
|  | Reverse | | CTGGCTTGGTCACATCTTG |  |
| CollagenI | Forward | | GGTGCCCCTGGAGAGAAT | 151 |
|  | Reverse | | GGACCAGCAGACCCAATG |  |
| E-cadherin | Forward | | actttggtgtgggtctggag | 214 |
|  | Reverse | | tctgtggcaatgatgagagc |  |
| Snail1 | Forward | | tcgcagtgagtggagagatg | 247 |
|  | Reverse | | agtcgggcttgctttctaca |  |
| Vimentin | Forward | | attgcaggagctgaatgacc | 150 |
|  | Reverse | | tccctcatctcctcctcgta |  |
| FSP1 | Forward | | ctgaaccctggttggcttta | 198 |
|  | Reverse | | atgaggacccaagtgacagg |  |
| α-SMA | Forward | | agggatcctgaccctgaagt | 219 |
|  | Reverse | | tacatggcagggacattgaa |  |
|  |  |  | |  |
